# Genetic variants of the class A scavenger receptor gene are associated with coronary artery disease in Chinese

**DOI:** 10.7555/JBR.26.20110116

**Published:** 2012-09-28

**Authors:** Min Zhang, Yan Zhang, Shuaishuai Zhu, Xiaoyu Li, Qing Yang, Hui Bai, Qi Chen

**Affiliations:** a Atherosclerosis Research Center, Key Laboratory of Cardiovascular Disease and Molecular Intervention, Institute of Reproductive Medicine, Nanjing Medical University, Nanjing, Jiangsu 210029, China;; b Department of Medicine, China-Japan Friendship Hospital, Beijing 100029, China.

**Keywords:** class A scavenger receptor gene, single nucleotide polymorphisms (SNPs), coronary artery disease, Chinese population

## Abstract

The class A scavenger receptor, encoded by the macrophage scavenger receptor 1 (*MSR1*) gene, is a pattern recognition receptor (PPR) primarily expressed in macrophages. It has been reported that genetic polymorphisms of *MSR1* are significantly associated with the number of diseased vessels and coronary artery narrowing greater than 20% in Caucasians. However, whether it links genetically to coronary artery disease (CAD) in Chinese is not defined. Here, we performed an independent case-control study in a Chinese population consisting of 402 CAD cases and 400 controls by genotyping ten single nucleotide polymorphisms (SNPs) of *MSR1*. We found that rs416748 and rs13306541 were significantly associated with an increased risk of CAD with per allele odds ratio (OR) of 1.56 [95% confidence interval (CI) = 1.28-1.90; *P* < 0.001] and 1.70 (95% CI = 1.27-2.27; *P* < 0.001), respectively. Our results indicate that genetic variants of *MSR1* may serve as predictive markers for the risk of CAD in combination with traditional risk factors of CAD in Chinese population.

## INTRODUCTION

Coronary artery disease (CAD) is one of the major causes of death in most countries, including China[1]. Genetic, behavioral, and environmental factors contribute to CAD where inflammatory responses play key roles. Inflammation is involved in early atherosclerotic lesion formation and unstable plaque rupture that would lead to the occurrence of CAD[2]. The class A scavenger receptor (SR-A) is a pattern recognition receptor (PRR) primarily expressed in macrophages. It participates in inflammation, oxidative stress, innate and adaptive immunity, and apoptosis by recognizing a broad spectrum of polyanionic ligands including Gram positive and negative bacteria, oxidized low-density lipoprotein (LDL), and silica[3]. The intracellular coupler-regulated property and the multiple endocytic routes suggest a regulatory role of SR-A in pathophysiologic microenrivoments[4]. In atherogenesis, SR-A plays an important role in mediating foam cell formation, cell apoptosis, and secretion of inflammatory products[5]. Recently, SR-A has been found to impact on macrophage polarization in ischemic heart injury[6].

SR-A is encoded by the gene macrophage scavenger receptor 1 (*MSR1*) that is located on chromosome 8p22. *MSR1* is composed of 11 exons and 10 introns. It can generate at least three protein isoforms by alternative RNA splicing, two functional isoforms (type I and type II) and one nonfunctional isoform (type III)[7],[8]. Genetic polymorphisms of *MSR1* have been identified to be associated with prostate cancer[9]–[11] and with coronary atherosclerosis burden in Caucasians[12]. Increased expression of *MSR1* in peripheral blood mononuclear cells has been found in patients with acute coronary syndrome. In human atherosclerotic lesions, *MSR1* expression is upregulated in fatty streaks and is downregulated in advanced complicated lesions[13],[14]. These results suggest that *MSR1* could be a predictive marker for a recurrence of a cardiovascular event[15]. In this study, we sought to test whether genetic variants in the *MSR1* gene alter susceptibility to CAD in a Chinese population. Our findings demonstrated that genetic variants of *MSR1* may serve as markers to predict the risk of CAD in Chinese.

## SUBJECTS AND METHODS

### Subjects

A total of 402 consecutive and unrelated CAD patients who were admitted to the First Affiliated Hospital of Nanjing Medical University due to suspected CAD were recruited from the inpatient department. The diagnosis of CAD was certified by coronary angiography with the Judkins technique using a quantitative coronary angiographic system[16]. The coronary angiograms were reviewed by experienced cardiologists who were unaware if the patients would be recruited into this study. CAD was diagnosed by angiographic evidence of > 50% organic stenosis in at least one segment of a major coronary artery including the left anterior descending, left circumflex, or right coronary artery.

Additional 400 patients were also admitted to this hospital as the control group. They were selected and matched by age (±5 years) and sex. Considering that it was unethical to perform coronary angiography to rule out the presence of asymptomatic CAD, the following inclusion criteria were used for enrollment of controls: the subjects had no history of angina and no symptoms or signs of other atherosclerotic vascular diseases.

All subjects enrolled in this study were Han Chinese and residing in or near Jiangsu province. They had no history of significant concomitant diseases, including cardiomyopathy, renal failure, bleeding disorders, previous thoracic irradiation therapy, and malignant diseases. Hypertension was defined as resting systolic blood pressure > 140 mmHg and/or diastolic blood pressure > 90 mmHg or in the presence of active treatment with antihypertensive agents. Individuals who smoked one cigarette per day for over one year were considered as smokers.

This study was approved by the Ethics Committee of the First Affiliated Hospital of Nanjing Medical University and informed consent was obtained from each participant.

### Blood sampling and extraction of DNA

A 5 mL peripheral venous blood sample was obtained from all participants. Part of this blood sample was analyzed for plasma levels of glucose, triglycerides (TG), total cholesterol, HDL-cholesterol (HDL-C), and LDL-cholesterol (LDL-C), all of which were measured using an automated chemistry analyzer (Olympus Au2700, Japan). Genomic DNA was extracted using the Blood Genome DNA Extraction Kit purchased from Takara Biotech Co. (Japan).

### SNP selection

Polymorphisms were selected by an approach combining both tagging SNPs and potentially functional SNPs of the *MSR1* gene. Tagging SNPs (tSNP) were chosen from genotyped SNPs of Chinese Han Beijing (CHB) in the HapMap database (minor allele frequency≥0.05, Hardy-Weinberg equilibrium *P*≥ 0.05) in HaploView 4.1 software on the basis of pairwise linkage disequilibrium (r^2^ threshold = 0.8) and with a priority of forcing the potentially functional SNPs in the evaluation. Potentially functional polymorphisms were identified to meet the following criteria: (a) located in the 5′-flanking regions, 5′-UTR, 3′-UTR, and coding regions with amino acid changes; (b) were shown to be of biological significance according to the literature review; (c) were associated with the gene expression and/or disease risk in previous studies. Among the ten SNPs selected, eight SNPs were successfully genotyped and consistent with those expected from the Hardy-Weinberg equilibrium (*P* > 0.05), whereas two SNPs (rs3747531 and rs33959637) that deviated from the Hardy-Weinberg equilibrium (*P* < 0.05) were removed from our further analysis.

### Genotyping

Genotyping of the seven SNPs (rs433235, rs11274081, rs33959637, rs3036811, rs12718376, rs4333601, and rs7840885) were performed using the PCR-LDR (polymerase chain reaction and ligase detection reaction) sequencing method, as reported previously[17],[18]. The PCR was carried out in a total volume of 15 µL containing 1.5 µL of 10×PCR buffer, 0.25 µL of each primer (10 pmol), 0.3 µL of dNTP, 0.25 µL of Taq polymerase (MBI Fermentas), 1 µL of genomic DNA, and 11.45 µL of H_2_O. The PCR cycling parameters were 35 cycles of 15 s at 94°C, 55°C for 15 s, and 72°C for 30 s. Ligase detection reaction (LDR) was performed in a total volume of 10 µL containing 3 µL of PCR product, 1 µL of 10×*Taq* DNA ligase buffer, 0.125 µL of 40 U/µL Taq DNA ligase (NEB), 0.01 µL 10 pmol probes (0.0033 µL each of probe), and 5.865 µL of H_2_O. LDR probes were composed of one common probe and two discriminating probes (designed by the Shanghai Generay Biotech Co., Shanghai, China). Subsequently, LDR products were analyzed by DNA sequencing (Model 377, Applied Biosystems). All assays were conducted blindly without the knowledge of case-control status.

Genotyping of the three SNPs (rs416748, rs13306541 and rs3747531) was performed using the TaqMan allelic discrimination assay on the platform of 7900HT Real-time PCR System (Applied Biosystems, Foster City, CA, USA). Two negative controls were included in each 384-well reaction plate and the genotyping results were determined by using SDS 2.3 Allelic Discrimination Software (Applied Biosystems). Moreover, to confirm the genotyping results, about 10% of the samples were randomly selected and retested by direct DNA sequencing on a 3730xl DNA analyzer (Applied Biosystems) and the accordance rate reached 100%.

### Statistical analysis

The Hardy-Weinberg equilibrium between SNPs was evaluated using the χ^2^ goodness-of-fit test among the control subjects. Two-sided χ^2^ tests were used to evaluate differences in the distributions of demographic characteristics, selected variables, and genotypes between the cases and controls. Logistic regression analyses were employed to estimate crude and adjusted odds ratios (ORs) and 95% confidence intervals (95% CIs) for the association between genetic variants and CAD risk in an additive model. The heterogeneity of association between subgroups was assessed using the χ^2^-based *Q* test. Linkage disequilibrium (LD) was estimated using the EH algorithm available online. We used the PHASE 2.0 program to infer the haplotype frequencies, based on the observed genotypes. *P*≤ 0.05 was considered statistically significant. All statistical analyses were performed with SPSS 13.0 (SPSS Inc., Chicago, IL, USA).

## RESULTS

### Demographic information

Characteristics of the 402 CAD cases and the 400 controls are shown in ***Table 1***. No significant differences were observed in age, sex, glucose, and TG between the cases and controls (*P* = 0.984, 0.784, 0.180, and 0.078, respectively). Compared with the control subjects, patients with CAD had higher levels of body mass index (BMI), TC, LDL-C, prevalence of hypertension, and rate of smoking, but lower HDL-C, all of which are established CAD risk factors.

### Association between *MSR1* polymorphisms and the risk of CAD

The observed genotype frequencies for rs416748, rs433235, rs13306541, rs11274081, rs3036811, rs12718376, rs4333601, and rs7840885 were all consistent with the Hardy-Weinberg equilibrium in controls (*P* = 0.079, 0.731, 0.067, 0.731, 0.066, 0.770, 0.873, and 0.511, respectively). Significant differences of genotype distributions between cases and controls were observed in rs416748 and rs13306541 (*P* < 0.001 for rs416748, and *P* = 0.002 for rs13306541), but not in rs433235, rs11274081, rs3036811, rs12718376, rs4333601, and rs7840885 (*P* = 0.445, 0.524, 0.961, 0.471, 0.357, and 0.401, respectively). As shown in ***Table 2***, logistic regression analysis revealed that individuals with variant alleles of rs416748 and rs13306541 were significantly associated with altered risk of CAD (adjusted per-allele OR=1.56, 95% CI = 1.28-1.90 for rs416748; adjusted per-allele OR=1.70, 95% CI=1.27-2.27 for rs13306541, respectively), with both *P* values < 0.001. However, no significant association was observed between the other six SNPs and risk of CAD. For consideration of false positivity, we used Bonferroni correction to further adjust the original *P* value. After Bonferroni correction, rs416748 is also significant using dominant genetic model (*P* after Bonferroni correction: 0.0034).

**Table 1:** Baseline characteristics of the study population

Variable	Case (*n* = 402)	Control (*n* = 400)	*P*-value
Age [year, *n*(%)]			< 0.984
< 60	146 (36.32)	145 (36.25)	
≥60	256 (63.68)	255 (63.75)	
Sex [*n*(%)]			< 0.784
Male	261 (64.93)	256 (64.00)	
Famle	141 (35.07)	144 (36.00)	
Smoking status [*n*(%)]			< 0.001
Yes	152 (37.81)	095 (23.75)	
No	250 (62.19)	305 (76.25)	
Hypertension [*n*(%)]			< 0.001
Yes	248 (61.69)	110 (27.50)	
No	154 (38.31)	290 (72.50)	
BMI (kg/m^2^)	24.87±2.940	23.34±3.070	< 0.001
Glucose (mmol/L)	5.46±1.45	5.34±1.12	< 0.180
TC (mmol/L)	4.83±1.03	4.33±1.24	< 0.001
TG (mmol/L)	1.74±1.04	1.62±0.87	< 0.078
HDL-C (mmol/L)	1.02±0.25	1.29±0.32	< 0.001
LDL-C (mmol/L)	2.83±0.77	2.56±0.93	< 0.001

BMI: body mass index; HDL-C: high density lipoprotein cholesterol; LDL-C: low density lipoprotein cholesterol; TC: total cholesterol; TG: triglyceride.

Age, TC, TG, HDL-C, LDL-C and glucose (expressed as mean±SD) were abnormally distributed and analyzed by Mann–Whitney U-test. BMI (expressed as mean±SD) was normally distributed and analyzed by Student's *t*-test. Other data were expressed as frequencies and percentages and evaluated by χ^2^-test.

**Table 2:** Distribution of genotypes of SNPs of *MSR1* and their associations with CAD risk

SNP	Genotype	Controls	Cases	Adjusted OR (95% CI)^a^	*P*-value^a^
		*n* (%)	*n* (%)		
rs416748	GG	159 (39.75)	101 (25.12)	1.00	
	AG	173 (43.25)	204 (50.75)	1.80 (1.26-2.58)	0.001
	AA	68 (17.00)	97 (24.13)	1.40 (1.12-1.76)	0.004
	Per allele			1.56 (1.28-1.90)	< 0.001
rs433235	AA	330 (82.50)	318 (79.10)	1.00	
	AG	66 (16.50)	78 (19.40)	1.18 (0.80-1.76)	0.409
	GG	4 (1.00)0	6 (1.50)0	1.39 (0.71-2.74)	0.341
	Per allele			1.24 (0.89-1.71)	0.199
rs13306541	AA	323 (80.75)	293 (72.89)	1.00	
	AG	69 (17.25)	83 (20.64)	1.27 (0.85-1.88)	0.244
	GG	8 (2.00)0	26 (6.47)0	1.97 (1.25-3.12)	0.004
	Per allele			1.70 (1.27-2.27)	< 0.001
rs11274081^b^	−−	330 (82.50)	319 (79.35)	1.00	
	+−	66 (16.50)	78 (19.40)	1.18 (0.79-1.76)	0.412
	++	4 (1.00)0	5 (1.25)0	1.32 (0.65-2.66)	0.444
	Per allele			1.21 (0.87-1.67)	0.260
rs3036811^c^	−−	129 (32.25)	126 (31.34)	1.00	
	+−	180 (45.00)	184 (45.77)	1.01 (0.71-1.44)	0.957
	++	91 (22.75)	92 (22.89)	1.02 (0.83-1.27)	0.823
	Per allele			1.02 (0.84-1.24)	0.834
rs12718376	TT	123 (30.75)	131 (32.59)	1.00	
	CT	195 (48.75)	179 (44.52)	0.77 (0.54-1.10)	0.154
	CC	82 (20.50)	92 (22.89)	0.98 (0.79-1.22)	0.866
	Per allele			1.01 (0.83-1.23)	0.912
rs4333601	GG	124 (31.00)	134 (33.33)	1.00	
	TG	196 (49.00)	177 (44.03)	0.74 (0.52-1.06)	0.101
	TT	80 (20.00)	91 (22.64)	0.98 (0.79-1.21)	0.820
	Per allele			1.01 (0.83-1.23)	0.951
rs7840885	GG	289 (72.25)	273 (67.91)	1.00	
	AG	100 (25.00)	117 (29.10)	1.35 (0.95-1.91)	0.092
	AA	11 (2.75)0	12 (2.99)0	0.91 (0.58-1.43)	0.675
	Per allele			1.18 (0.91-1.54)	0.216

a: Adjusted for age, sex, BMI, smoking and hypertension; b: “+” and “−” denote with and without the 15-bp sequence “TACTATAAATCATTC”, respectively; c: “+” and “−” denote with and without the 3-bp sequence “TAA”, respectively.

### Stratified analyses of the polymorphisms and CAD

Stratification analyses were conducted to evaluate the potential association of genetic variants of eight SNPs with the risk of CAD in subgroup populations. Variant allele of rs416748 (A) had stronger effect on females (adjusted OR=2.56, 95% CI=1.43−4.60, *P* = 0.002), subjects with BMI<24 (adjusted OR=2.34, 95% CI=1.38−3.99, *P* = 0.002), and no hypertension (adjusted OR=2.34, 95% CI=1.46−3.75, *P* < 0.001). The rs13306541-G allele had stronger effect on females (OR=3.26, 95% CI=1.40-7.59, *P* = 0.006), subjects with BMI<24 (adjusted OR=1.85, 95% CI=1.05−3.24, *P* = 0.032), and no hypertension (adjusted OR=1.70, 95% CI=1.01−2.85, *P* = 0.044). However, there was no obvious evidence of significant association between rs433235, rs11274081, rs3036811, rs12718376, rs4333601 and CAD risk among all subgroups.

We also conducted stratified analysis according to TC, TG, HDL-C, and LDL-C in dominant model, using the median in controls as cutoff values. Variant alleles of the eight SNPs had stronger effect on subjects with higher levels of TC, TG and LDL-C, but lower HDL-C.

### LD analysis of the polymorphisms and CAD

**Fig. 1:**
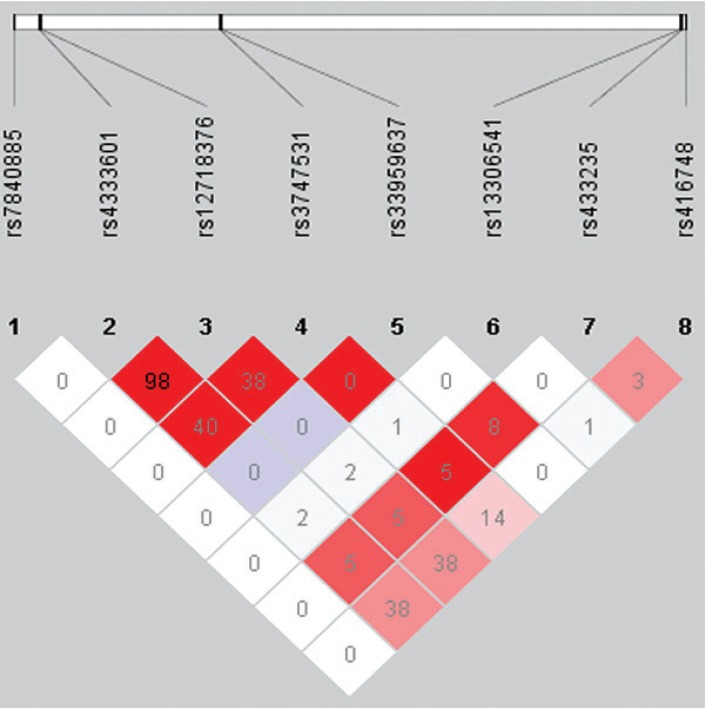
LD plot of the SNPs of MSR1.

In the LD analysis, two SNPs, rs11274081 and rs3036811, were structure variations, so they were not presented in the LD plot. In ***Fig. 1***, two SNPs, rs12718376 and rs4333601, were in high LD (r^2^ = 0.98, D' = 1.00). Thus, haplotype analysis was performed for these two SNPs. There were three haplotypes derived from the observed genotypes, but no significant difference was found in the distribution of haplotypes between cases and controls (***Table 3***).

### DISCUSSION

CAD is a complication of atherosclerosis. Occurrence and development of atherosclerosis is determined by many genetic and environmental factors. Genes involved in inflammation are potential candidates for screening the markers of atherosclerosis inheritance[19]. *MSR1* encodes an important PRR SR-A mediating lipid accumulation, apoptosis and production of cytokines in macrophages. All these macrophage activities are prerequisites for atherogenesis. We demonstrated for the first time that genetic variants of *MSR1* are significantly associated with CAD in Chinese population.

**Table 3:** Haplotype analysis of rs12718376 and rs4333601 in patients with CAD and unaffected control subjects

Haplotypes		Cases	Controls	OR (95% CI)	z	*P* > |z|
rs12718376	rs4333601	(*n* = 800)	(*n* = 804)			
T	G	441	441	1.00		
C	T	356	359	0.99 (0.80-1.21)	-0.12	0.90
C	G	3	4	1.50 (0.32-7.01)	-0.52	0.61

We evaluated the associations of ten genetic variants of *MSR1* with CAD susceptibility in an independent case-control study with 402 CAD cases and 400 controls in a Chinese population. Each of these ten genetic variants could have an important impact on *MSR1* function. For example, the SNPs in the promoter region of rs416748, rs433235, and rs13306541 could affect transcription of the *MSR1* gene. The missense change of rs3747531 could affect *MSR1* binding with its ligands, because it changes a conserved residue in the first Gly-X-Y repeat of the collagenous domain of the protein, which is critical for ligand binding[11],[12]. The exon 9 of *MSR1* encodes the carboxyl-terminal domain of type II isoform, which is responsible for recognizing the extracellular signals. Therefore, these two missense changes, rs4333601 and rs12718376, may be involved in *MSR1*'s functions in responding to external stimulation. The rs11274081, rs33959637, and rs3036811 are noncoding SNPs located in intron 1, 5, and 7 of the gene, respectively, and thus do not likely directly predict the alternations in protein functions. Alternatively, they might play a role in posttranslational adaptation mechanisms and affect mRNA levels or splicing. We found that rs416748 and rs13306541 but not rs433235, rs11274081, rs3036811, rs12718376, rs4333601, and rs7840885 were significantly associated with increased risk of CAD in the study cohort. The effect appeared to be stronger in females, and subjects with BMI < 24 and no hypertension.

Logistic regression analysis supported the hypothesis that rs416748 and rs13306541 may contribute to CAD susceptibility in our population. Both SNPs showed the potential to predict the risk of CAD in future in combination with traditional risk factors of CAD in Chinese population. They may be used to build the risk prediction model to serve as the genetic test for high risk population of CAD. Both SNPs are located in the promoter region that is responsible for transcription of the *MSR1* gene. Thus, they may influence the expression level of this gene. Nakayama et al. have found that the *MSR1* gene expression level in peripheral blood mononuclear cells (PBMCs) is increased in patients with acute coronary syndrome (ACS), indicating that the *MSR1* gene expression level in cells also provides a predictive marker for a re-attack of a cardiovascular event[15]. It is plausible that individuals may differ in the extent of inflammatory response to modified LDL, depending on the *MSR1* genotype. Genetic variations of *MSR1* may affect the extent of the inflammatory process culminating in different lesion sizes, despite a similar modified LDL load. These findings imply a significant pathophysiological effect beyond lipid accumulation in cells for the *MSR1* gene in the development of atherosclerosis in humans.

Prior investigators have genotyped three polymorphic sites in the *MSR1* gene including rs3036811, rs33959637, and rs3747531 in 136 non-diabetic Ashkenazi men under the age of 55 years undergoing coronary angiography. They found significant associations between rs33959637 and the number of diseased vessels and coronary artery narrowing greater than 20%, which remained significant upon controlling for age, cholesterol level, hypertension, and smoking[12]. However, this SNP was found to deviate from the Hardy-Weinberg equilibrium in our study, with only two genotypes (CC and AC). These differences may come from the different experimental conditions, ethnicity variation, and exposure to different environmental factors.

Furthermore, in this independent case-control study, we failed to observe significant associations of rs433235, rs11274081, rs3036811, rs12718376, rs4333601, and rs7840885 with the risk of CAD. The small sample size may account for the negative results. The possibility may be that the association between these variants and CAD risk is very modest and our analysis would not have enough statistical power to detect it. Another explanation is that this association is not real in CAD. Larger sample size and well-organized studies are warranted to further clarify the associations of these SNPs with CAD.

In this study, we used candidate gene association studies on assumptions about biological relevant genes. Despite some successes, single gene association studies remain problematic. Small scale studies are easy to yield a false positive association. There is evidence of publication bias in the literature that might enrich false positives[20],[21]. With improved genotyping technologies, genome-wide association studies (GWASs) have recently become an important approach in genetic studies. GWASs are large scale association mapping using SNPs, making no assumptions of the genomic location or function of the causal variant and are an unbiased and fairly comprehensive option that can be attempted even in the absence of convincing evidence regarding function or location of the causal genes[22]. Recent GWASs and meta-analysis in CAD identified several new susceptibility loci[23]–[26]. Therefore, large scale association studies are warranted to further validate our results.

One of the strengths in the current study is that, based on an independent Chinese study, we firstly confirmed the *MSR1* gene as a susceptibility gene for CAD in Chinese population. However, the limitations of this study need to be addressed. First, although great attention was paid in the study design and analysis, selection bias in the present study might have affected our results. Second, the relatively small sample size may underpower the results of our study. Third, all our data were obtained at the time of diagnosis, thus prospectively followed-up clinical outcome including severe cardiac events may be required to analyze the association between the genetic variants of *MSR1* and CAD prognosis. Last, our study was performed only in a Chinese population. Data should be extrapolated to other regions and ethnic groups cautiously.

In conclusion, this study provided strong evidence in an independent population that genetic variants of *MSR1*, defined by rs416748 and rs13306541, respectively, were susceptible SNPs for CAD in Chinese population. The functional significance of the variants remains to be further investigated, which may help in elucidating the genetic mechanism of CAD.
